# The Plus End-Directed Microtubule (Kinesin-3 Family) Motor Protein KIF13B Is Associated with the Photoreceptor Synaptic Ribbon Complex

**DOI:** 10.3390/ijms26136044

**Published:** 2025-06-24

**Authors:** Shweta Suiwal, Karin Schwarz, Stephan Maxeiner, Frank Schmitz

**Affiliations:** Institute of Anatomy and Cell Biology, Medical School Homburg, Saarland University, 66421 Homburg, Germany; karin.schwarz@uks.eu (K.S.); stephan.maxeiner@uni-saarland.de (S.M.)

**Keywords:** retina, photoreceptor, ribbon synapses, KIF13B, kinesin-3, synaptic ribbon, RIBEYE, active zone

## Abstract

Retinal ribbon synapses are continuously active chemical synapses. The eponymous synaptic ribbon is anchored to the active zone neurotransmitter release sites of ribbon synapses, recruits synaptic vesicles and guides ribbon-associated synaptic vesicles to the release sites. RIBEYE is the major protein component of synaptic ribbons. But likely, additional proteins contribute to ribbon synapse function. The synaptic ribbon of photoreceptor synapses is embedded into a highly polarized microtubule cytoskeleton. Interestingly, proteins of the photoreceptor primary cilium, such as NPHP4 and other ciliary proteins, including KIF3A, were shown to be localized to photoreceptor synaptic ribbons. Previous studies demonstrated that the microtubule motor protein KIF13B catalyzes secretory vesicle transport to the plus ends of microtubules and identified an interaction of KIF13B with NPHP4 at primary cilia. However, the localization of KIF13B, a kinesin-3 family motor protein, in the retina is still unknown. In the present study, we used two different antibodies against KIF13B and high-resolution confocal microscopy, super-resolution structured illumination microscopy (SR-SIM), and post-embedding immunogold electron microscopy to determine the localization of KIF13B in retinal photoreceptors. Apart from its localization at the primary photoreceptor cilium, we found a strong enrichment of KIF13B at photoreceptor synaptic ribbons. The synaptic ribbon is needed for the synaptic enrichment of KIF13B as shown by analyses of synaptic ribbon-deficient RIBEYE knockout mice. These findings suggest that KIF13B performs vesicle trafficking functions at the photoreceptor synaptic ribbon complex at the interface between the synaptic ribbon and the presynaptic microtubule transport system.

## 1. Introduction

Ribbon synapses in the retina, pineal gland and hair cells of the inner ear are specialized for continuous synaptic transmission [1,2,3]. Ribbon synapses in the retina are made by photoreceptors and bipolar cells [2,3,4]. Photoreceptors possess a bipolar morphology with two distinct processes. The distal process, which contacts the retinal pigment epithelium (RPE), is the light-transducing outer segment [5,6,7]. The outer segment is connected to the inner segment via a thin bridge segment that harbors a microtubule-based primary cilium, the connecting cilium (CC) [8,9,10,11,12,13]. The connecting cilium controls the import and export of proteins into/from the outer segment (OS), e.g., the import/export of proteins involved in phototransduction and/or light-/dark-adaptation [7,10,12,14,15,16].

The proximal photoreceptor process forms the presynaptic terminal that continuously transmits sensory information to the inner retina at the outer plexiform layer (OPL) [1,2,3,17]. The presynaptic terminals contain synaptic ribbons to promote continuous synaptic transmission [1,2,3,18]. Synaptic ribbons are anchored to the active zone neurotransmitter release sites and bind large numbers of synaptic vesicles that are delivered to the active zone for continuous exocytosis and neurotransmitter release [19,20,21,22]. The main component of synaptic ribbons is the RIBEYE protein [3,23,24,25,26,27]. RIBEYE is essential for the making of synaptic ribbons because the deletion of RIBEYE leads to a complete absence of synaptic ribbons in the retina [26,28] and inner ear [29,30].

Apart from the fact that RIBEYE is essential to build the synaptic ribbon, the full protein composition of photoreceptor synaptic ribbons is incompletely understood. The active zone proteins Bassoon and Piccolo/Piccolino were shown to contribute to synaptic ribbon architecture in photoreceptor synapses [31,32,33,34,35]. Interestingly, previous studies indicated that the photoreceptor primary cilium and the synaptic ribbon share some common proteins that might enable similar functions at these different sub-cellular sites. Won et al. [36] demonstrated that the ciliary protein NPHP4 is associated with photoreceptor synaptic ribbons, similar to KIF3A, a canonically anterograde kinesin-2 family microtubule motor protein also present at the photoreceptor primary cilium [37,38]. A recent study revealed that another set of ciliary proteins involved in the trafficking of myristoylated proteins is also associated with photoreceptor synaptic ribbons [39].

NPHP4 interacts with the plus-end-directed microtubule motor KIF13B, a kinesin-3 family motor protein [40]. KIF13B (also called GAKIN, for guanylate kinase-associated kinesin) is an anterograde, plus-end-directed microtubule motor protein [38,41,42,43,44,45,46,47]. The kinesin-3 microtubule motor protein KIF13B promotes the transport of vesicles, including secretory vesicles, to the fast growing plus ends (“+TIPs”) of microtubules [48,49,50,51,52]. A recent study demonstrated that KIF13B is also involved in bidirectional movement at the primary cilia of immortalized human retinal pigment epithelial hTERT-RPE1 cells [53].

Photoreceptors possess a highly polarized microtubule cytoskeleton, and a microtubule network is present close to the synaptic ribbons in the retina and the inner ear [37,54,55,56,57]. In the presynaptic photoreceptor terminals, microtubules end with their fast-growing plus-end, the “+TIP” [37,54,57]. In the current study, we aimed to localize the motor protein KIF13B in the retina particularly concerning its localization in photoreceptor cells. Tissue expression databases indicated the expression of KIF13B in the retina (www.proteinatlas.org/ENSG00000197892-KIF13B/brain/retina, accessed on 28 April 2025; [58,59]). In our morphological analyses, we focused on photoreceptor cells of the retina. In retinal photoreceptors, the primary cilium and the presynaptic ribbon terminal are morphologically clearly defined and spatially well separated from each other so that these compartments can be unambiguously identified [60]. For the immunolocalization of KIF13B, we employed two independent KIF13B antibodies and applied confocal microscopy, super-resolution structured illumination microscopy (SR-SIM) and post-embedding immunogold electron microscopy to analyze the sub-cellular localization of KIF13B. Our study demonstrated that kinesin motor protein KIF13B is highly enriched at photoreceptor synaptic ribbons suggesting that the KIF13B microtubule motor protein is involved in synaptic ribbon-associated vesicle trafficking possibly in a similar fashion as at the photoreceptor primary cilium.

## 2. Results

In the present study, we analyzed the distribution of kinesin-3 family motor protein KIF13B in photoreceptor synapses of the outer plexiform layer (OPL) of the mouse (and bovine) retina. For our analyses, we used two independent KIF13B antibodies, a commercially available antigen-affinity purified rabbit polyclonal KIF13B antibody and a newly generated mouse monoclonal KIF13B antibody (clone 5C10).

**Figure 1:**
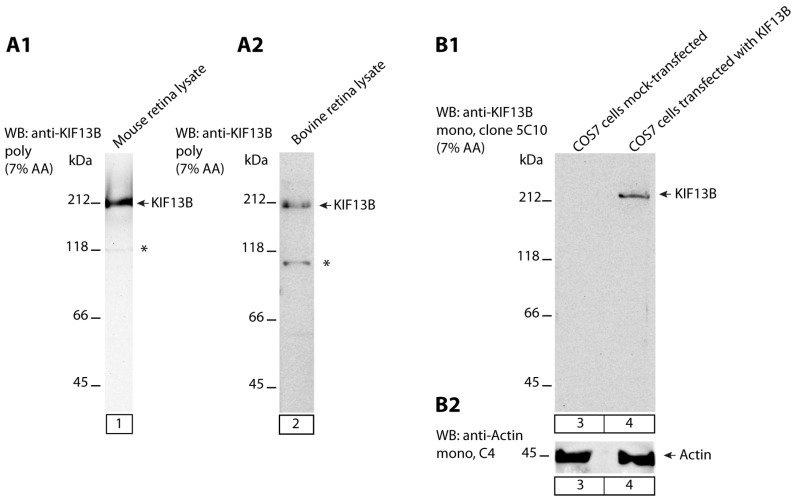
KIF13B expression in the retina. (**A1**,**A2**) KIF13B expression was characterized with lysates obtained from wild-type mouse retinas (**A1**) and bovine retinas (**A2**) by Western blot (WB) with affinity-purified rabbit polyclonal KIF13B antibody. A strong major high molecular weight band at ≈200 kDa, the expected running position of full-length KIF13B, was observed in the retinal lysates (**A1**,**A2**). A weaker second, lower molecular weight band (marked by an asterisk) could represent a degradation product of KIF13B. (**B1**,**B2**) WB analyses of KIF13B expression in transfected COS7 cells probed with mouse monoclonal KIF13B antibody (clone 5C10). Lysates from COS7 cells transfected with a full-length KIF13B (1843 amino acid)-encoding plasmid was loaded in lane 4; lysate from mock-transfected control COS7 cells in lane 3. Lanes 3 and 4 were first probed with mouse monoclonal anti-KIF13B (clone 5C10) (**B1**). Afterwards, the same blot was re-probed after stripping with anti-Actin for verification of equal total protein loading (**B2**). Abbreviation: 7% AA, 7% acrylamide gel; poly, polyclonal; mono, monoclonal.

First, we characterized the specificity of the KIF13B antibodies by Western blot (WB). In WB analyses, the KIF13B rabbit polyclonal antibody detected a strong major band at the expected running position of ≈200 kDa in lysates from mouse and bovine retinas (Figure 1(A1,A2)). Similarly, the mouse monoclonal KIF13B antibody clone 5C10 detected a KIF13B band only in COS7 cells that were transfected with the full-length KIF13B-encoding eukaryotic expression plasmid but not in mock-transfected COS7 cells (Figure 1(B1)). Actin served as a loading control in these experiments (Figure 1(B2)) to verify equal protein loading of the respective cell lysates. We tested the mouse monoclonal KIF13B antibody (clone 5C10) in WB experiments with transfected COS7 cells because the abundance of KIF13B in retinal lysates was too low to be detected by the monoclonal KIF13B antibody (clone 5C10).

**Figure 2:**
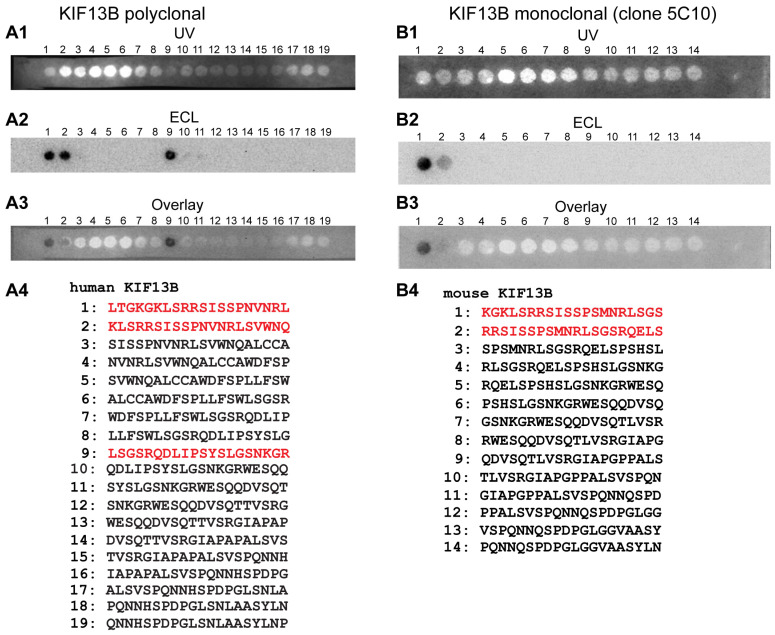
Epitope mapping of the mono- and polyclonal KIF13B antibodies with overlapping multi-peptide arrays. (**A1**–**A4**) The indicated overlapping multi-peptide array that covers the protein region used for immunization/generation of the polyclonal rabbit antibody was probed with the rabbit polyclonal KIF13B antibody by Western blot (WB). (**A1**) UV illumination of the peptide arrays visualizes the position of the respective peptide spots. (**A2**) shows the WB result and (**A3**) is the overlay of (**A1**,**A2**). (**B1**–**B4**) The indicated overlapping multi-peptide array was probed with the KIF13B mouse monoclonal antibody clone 5C10 to determine the precise binding epitope of the monoclonal antibody. (**B1**) UV illumination of the peptide arrays visualizes the position of the respective peptide spots. (**B2**) shows the WB result and (**B3**) is the overlay of (**B1**,**B2**). (**A4**,**B4**) Amino acid sequences of the indicated peptide spots. The peptide sequences of peptide spots that strongly reacted with the polyclonal KIF13B antibody and monoclonal KIF13B antibody clone 5C10 are highlighted in red.

To further characterize the KIF13B antibodies, we mapped the precise epitopes (peptide binding sites) of the KIF13B polyclonal and monoclonal antibodies with overlapping multi-peptide arrays (Figure 2). Overlapping multi-peptide arrays, that encompass the full KIF13B protein region against which the respective KIF13B antibodies were raised, were probed by Western blot with the respective KIF13B monoclonal or polyclonal antibody to map their respective binding sites (Figure 2). Each of the individual peptide spots of the overlapping multi-peptide arrays contained a 20 amino acid long peptide of the respective KIF13B protein region used for immunization.

The KIF13B peptide sequences detected by the KIF13B rabbit polyclonal antibody (i.e., LTGKGKLSRRSISSPNVNRLSVWNQ/LSGSRQDLIPSYSLGSNKGR; highlighted in red in Figure 2(A4)) are highly conserved in mice and cattle (90.5% identity in mice, XP_006518685.1; 100% in cattle; XP_061282784.1/85% in mice; XP_006518685.1 and 90% in cattle; XP_061282784.1). Similarly, the mouse KIF13B peptide sequence detected by the KIF13B mouse monoclonal antibody clone 5C10 (KGKLSRRSISSPSMNRLSGSRQELS; highlighted in red in Figure 2(B4)) is also highly conserved in humans as well as in cattle (87.5% identity in both species, NP_056069.2 and XP_061282784.1).

**Figure 3:**
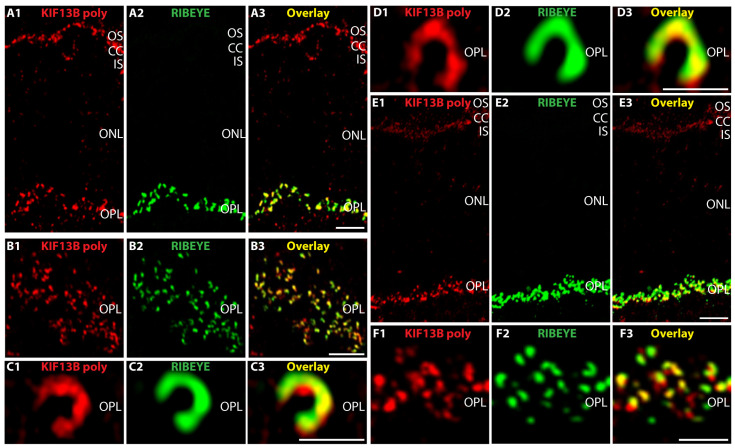
KIF13B is strongly enriched at synaptic ribbons of photoreceptor synapses in the OPL. (**A**–**D**) Cryostat sections of the bovine retina double-immunolabeled with affinity-purified rabbit polyclonal antibody against KIF13B (red channel, (**A1**,**B1**,**C1**,**D1**)) and mouse anti-RIBEYE(B)-domain/CtBP2 (clone 2D9) (green channel, (**A2**,**B2**,**C2**,**D2**)). Overlay is shown in the yellow channel (**A3**,**B3**,**C3**,**D3**). Please note that under the selected conditions (native, flash-frozen, non-PFA fixed cryostat sections), the antibody against RIBEYE(B)-domain (clone 2D9) strongly labels RIBEYE at synaptic ribbons. Under these conditions, the focal concentration of CtBP2 in the photoreceptor nuclei is too low to become detectable by IF. (**E**,**F**) Cryostat sections of the mouse retina double-immunolabeled with affinity-purified rabbit polyclonal antibody against KIF13B (red channel, (**E1**,**F1**)) and mouse anti-RIBEYE (clone 2D9) (green channel, (**E2**,**F2**)). Overlay of the red channels (**E1**,**F1**) and green channels (**E2**,**F2**) is shown in (**E3**,**F3**). With the KIF13B polyclonal antibody, a strong punctate, horseshoe-shaped KIF13B immunosignal in the OPL was observed that largely overlapped with photoreceptor synaptic ribbons that were visualized by immunolabeling with RIBEYE (clone 2D9) antibody. (**A**,**B**,**E**,**F**) were obtained by confocal microscopy; (**C**,**D**) by super-resolution structured illumination microscopy (SR-SIM). Abbreviations: OS, outer segment; CC, connecting cilium; IS, inner segment; ONL, outer nuclear layer; OPL, outer plexiform layer; IF, immunofluorescence; poly, polyclonal. Scale bars: 5 µm (**A**,**B**,**E**,**F**); 1 µm (**C**,**D**).

Next, we immunolabeled cryostat sections of the mouse and bovine retina with the polyclonal KIF13B antibody together with mouse monoclonal antibody against RIBEYE (clone 2D9; directed against RIBEYE(B)-domain/CtBP2) to visualize synaptic ribbons (Figure 3). We found very similar results in the immunolabeled mouse and bovine retina (Figure 3). We found KIF13B highly enriched in the outer plexiform layer (OPL) of the retina where the photoreceptor synapses are located. In the OPL, the KIF13B immunosignals largely overlapped with the synaptic ribbons which were immunolabeled with the mouse monoclonal RIBEYE antibody (clone 2D9; [28,61]). The polyclonal KIF13B antibody showed strong discrete/punctate immunosignals close to the RIBEYE-immunolabeled synaptic ribbons (Figure 3A,B). In addition to the strong immunolabeling of the synaptic ribbons in the OPL, we also observed KIF13B immunosignals at the connecting cilium which connects the photoreceptor outer and inner segment (Figure 3A). The KIF13B immunolabeling of the connecting cilium is expected because KIF13B is a component of primary cilia [40,53] to which also the connecting cilia of photoreceptors belong.

Super resolution-structured illumination microscopy (SR-SIM) of the KIF13B-immunolabeled structures in the OPL revealed a horseshoe-shaped appearance of the KIF13B immunosignals (Figure 3C,D). A horseshoe-shaped immunolabeling pattern is characteristic for immunolabeled photoreceptor synaptic ribbons [3,23,62]. In support of this notion, the KIF13B co-localized with the immunosignals of RIBEYE, the major component of synaptic ribbons in SR-SIM analyses (Figure 3C,D). These analyses demonstrated that motor protein KIF13B is highly enriched close to photoreceptor synaptic ribbons. SR-SIM imaging also indicated that some parts of the photoreceptor synaptic ribbon show particularly strong KIF13B immunosignals while other parts of the photoreceptor synaptic ribbon show a less intense KIF13B immunosignal (Figure 3C,D) indicating the presence of KIF13B “hotspots” at photoreceptor synaptic ribbons. These hotspots might represent sites at the synaptic ribbon where the +TIPs of microtubules are anchored close to the synaptic ribbon (see discussion). Unfortunately, the KIF13B rabbit polyclonal antibody was not suitable to ultrastructural analyses.

Therefore, we generated a novel monoclonal antibody against KIF13B, i.e., the KIF13B mouse monoclonal antibody clone 5C10, to answer this question. In Western blots, The KIF13B (clone 5C10) monoclonal antibody detected a single band at the expected running position of ≈200 kDa in COS7 cells that were transfected with a full-length KIF13B-encoding plasmid but not in mock-transfected COS7 cells (Figure 1). The precise epitope of KIF13B (clone 5C10) antibody was determined by WB with the overlapping peptide arrays that covered the peptide used for immunization (Figure 2(B1–B4)).

**Figure 4:**
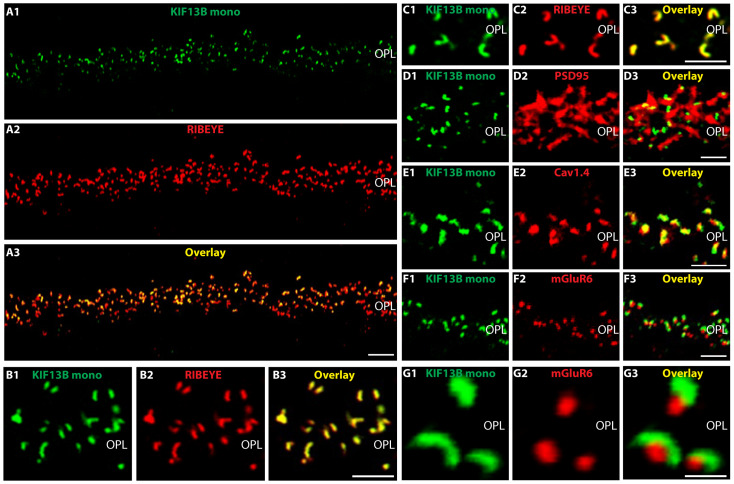
KIF13B is strongly enriched at photoreceptor synaptic ribbons in the outer plexiform layer. (**A**–**C**) Cryostat section of the mouse retina double-immunolabeled with mouse monoclonal antibody against KIF13B (clone 5C10) and rabbit polyclonal antibody against RIBEYE (U2656). (**B**,**C**) The KIF13B antibody (green channel) generates a discrete, horseshoe-shaped immunolabeling pattern in the OPL, which is highly enriched at the synaptic ribbon, as judged by co-localization with RIBEYE (red channel). (**D**–**G**) Mouse retina cryosections double-immunolabeled with mouse monoclonal KIF13B (clone 5C10) (**D**–**G**) and rabbit polyclonal anti-PSD95 (**D2**), rabbit polyclonal antibody against Cav1.4 C-term (**E2**) and rabbit polyclonal antibody against mGluR6 (**F2**,**G2**). Signals from red channels (**A2**,**B2**,**C2**,**D2**,**E2**,**F2**,**G2**) and green channels (**A1**,**B1**,**C1**,**D1**,**E1**,**F1**,**G1**) were merged in (**A3**,**B3**,**C3**,**D3**,**E3**,**F3**,**G3**). Abbreviations: OPL, outer plexiform layer; mono, monoclonal. Scale bars: 5 µm (**A**–**F**), 1 µm (**G**).

We used this novel monoclonal KIF13B antibody (clone 5C10) for the immunolocalization of KIF13B in the retina at the light- and electron microscopic level. Using light microscopical immunolabeling with the novel KIF13B monoclonal antibody, we observed a strong KIF13B immunosignal in the outer plexiform layer (OPL) in which the photoreceptor synapses are located (Figure 4A–G). The KIF13B immunosignal obtained with the novel KIF13B monoclonal antibody was horseshoe-shaped, similarly as observed with the rabbit polyclonal KIF13B antibody and largely overlapped with the RIBEYE immunosignals (Figure 4A–C). Thus, the immunosignals obtained with the monoclonal KIF13B antibody were qualitatively similar to the immunolabeling results obtained with the polyclonal KIF13B antibody.

We performed additional double-immunolabeling experiments with the mouse monoclonal KIF13B antibody (clone 5C10) to further characterize KIF13B localization in photoreceptor synapses (Figure 4D–G). KIF13B was found close to the presynaptic active zone neurotransmitter release sites that were labeled with antibodies against voltage-gated Cav1.4 channels (Figure 4E). Similarly, KIF13B immunosignals were found within the PSD95-immunolabeled presynaptic photoreceptor terminals (Figure 4D) but did not co-localize with the postsynaptic marker mGluR6, which is enriched at the postsynaptic tips of invaginating bipolar cells [63,64,65] (Figure 4F,G). In conclusion, the mouse monoclonal KIF13B (clone 5C10) antibody also confirmed the localization of KIF13B at photoreceptor synaptic ribbons at the light microscopical level (Figure 4), similar to what was also observed with the rabbit polyclonal KIF13B antibody (Figure 3).

**Figure 5:**
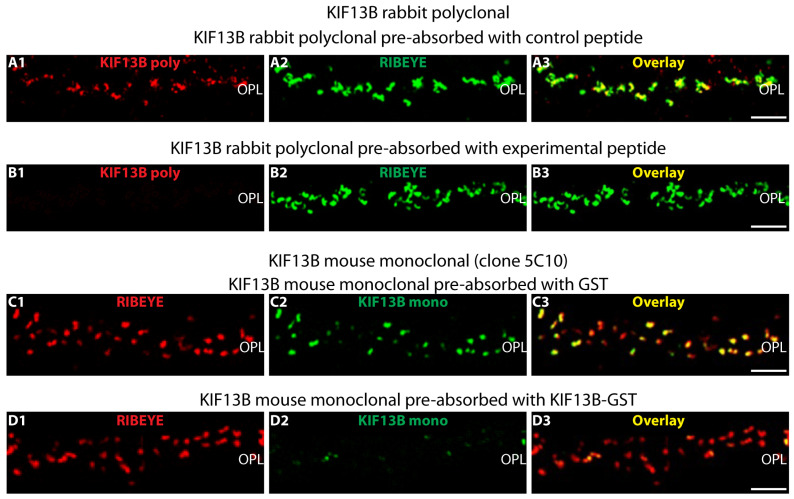
(**A**–**D**) Validation of the KIF13B antibodies by pre-absorption. (**A1**–**A3**,**B1**–**B3**) Double immunolabeling of retina cryosections with the polyclonal KIF13B antibody (red channel) which was pre-absorbed with either KIF13B-HIS-tagged fusion protein (**B1**) or with an unrelated, HIS-tagged control fusion protein (**A1**). The sections were double-immunolabeled with monoclonal RIBEYE antibody (clone 2D9) (green channel, (**A2**,**B2**)) for visualization of the synaptic ribbons that served as a reference structure. Overlay of the red channel (**A1**,**B1**) and green channel (**A2**,**B2**) is shown in (**A3**,**B3**). (**C1**–**C3**,**D1**–**D3**). Confocal images of retina cryosections double-immunolabeled with mouse monoclonal KIF13B (clone 5C10) antibody (green channel) and with rabbit polyclonal anti-RIBEYE (U2656) antibody (red channel). The monoclonal KIF13B (clone 5C10) antibody was pre-absorbed with either the peptide, against which the KIF13B (clone 5C10) antibody was generated (**D2**), or an unrelated control peptide (**C2**). Signals from the red channels (**C1**,**D1**) and green channels (**C2**,**D2**) were merged in (**C3**,**D3**). Abbreviations: OPL, outer plexiform layer; poly, polyclonal; mono, monoclonal. Scale bars: 5 µm.

The specificity of KIF13B immunosignals at synaptic ribbon were verified by pre-absorption of both monoclonal KIF13B (clone 5C10) and polyclonal KIF13B antibody with their respective antigens. KIF13B immunosignals were absent in the immunolabeling of polyclonal KIF13B antibody pre-absorbed with KIF13B-HIS fusion protein (Figure 5(B1)). The pre-absorption with control HIS fusion protein has no impact on immunosignals of KIF13B in the OPL (Figure 5(A1)). Similarly, pre-absorption of the monoclonal KIF13B (clone 5C10) with KIF13B-GST fusion protein strongly reduced the KIF13B synaptic immunosignals (Figure 5(D2)) whereas no effect on KIF13B immunosignals was observed for pre-absorption with the control GST fusion protein (Figure 5(C2)). Pre-absorption experiment with KIF13B peptides/proteins had no effect on RIBEYE signals, indicating the specificity of the blocking experiments (Figure 5(A2,B2,C1,D1)).

**Figure 6:**
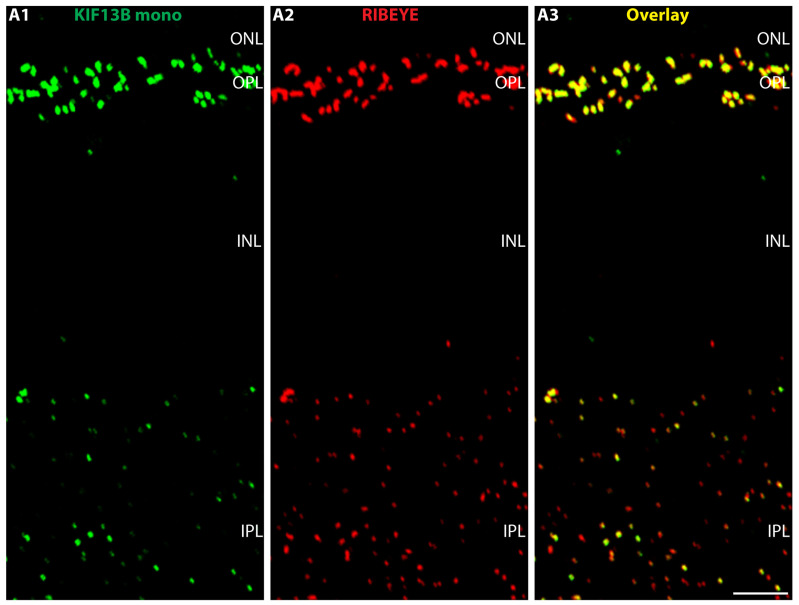
(**A1**–**A3**) KIF13B is present in the inner plexiform layer (IPL) of the retina close to synaptic ribbons. (**A**) Cryostat section of the mouse retina double-immunolabeled with mouse monoclonal antibody against KIF13B (clone 5C10) (green channel, (**A1**)) and rabbit polyclonal antibody against RIBEYE (U2656) (red channel, (**A2**)). The overlay image is shown in (**A3**). KIF13B immunosignals are present in the IPL close to the synaptic ribbons that were immunolabelled with anti-RIBEYE. KIF1B immunosignals in the IPL are typically weaker than in the OPL. Abbreviations: ONL, outer nuclear layer; OPL, outer plexiform layer; INL, inner nuclear layer; IPL, inner plexiform layer; mono, monoclonal. Scale bars: 5 µm.

KIF13B is present also in the inner plexiform layer (IPL) of the mouse retina but less strongly enriched than in the OPL as judged by double-immunolabeling with KIF13B mouse monoclonal antibody clone 5C10 and rabbit polyclonal antibody against RIBEYE (U2656) (Figure 6).

**Figure 7:**
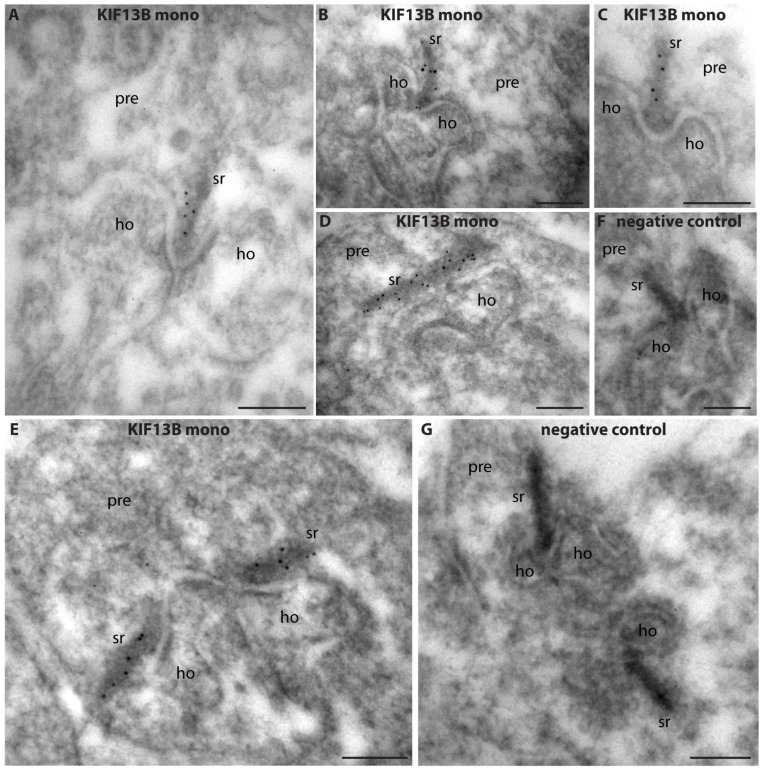
Post-embedding immunogold labeling of photoreceptor synapses with mouse monoclonal KIF13B antibody (clone 5C10). Ultrathin mouse retina LR Gold sections were immunolabeled with monoclonal anti-KIF13B (clone 5C10) and goat anti-mouse antibody conjugated to 5 nm gold particles (**A**–**E**). In the negative control incubations (**F**,**G**), all immunolabeling steps were the same except that the primary antibody incubations were omitted and replaced by incubations with blocking buffer. Abbreviations: sr, synaptic ribbon; pre, presynaptic; ho, dendritic tips of horizontal cells; mono, monoclonal. Scale bars: 200 nm.

Next, we determined the ultrastructural localization of KIF13B in rod photoreceptor ribbon synapses by using post-embedding immunogold electron microscopy and the monoclonal KIF13B antibody clone 5C10. Post-embedding immunogold labeling with monoclonal KIF13B (clone 5C10) revealed a strong enrichment of KIF13B immunogold particles at the synaptic ribbon in the presynaptic terminals of rod photoreceptor synapses in the outer plexiform layer (Figure 7). The entire synaptic ribbon was covered by KIF13B immunogold puncta (Figure 7A–E). No immunogold labeling was observed in the negative control incubations in which the primary antibody was omitted with all other steps remaining the same (Figure 7F,G).

The light- and electron microscopical immunolabeling data consistently demonstrated the strong enrichment of KIF13B at synaptic ribbons of rod photoreceptor ribbon synapses. Therefore, we investigated the impact of ablation of the synaptic ribbon on the synaptic localization of KIF13B. For this purpose, we made use of the RIBEYE knockout mice in which synaptic ribbons are completely absent [26,28].

**Figure 8:**
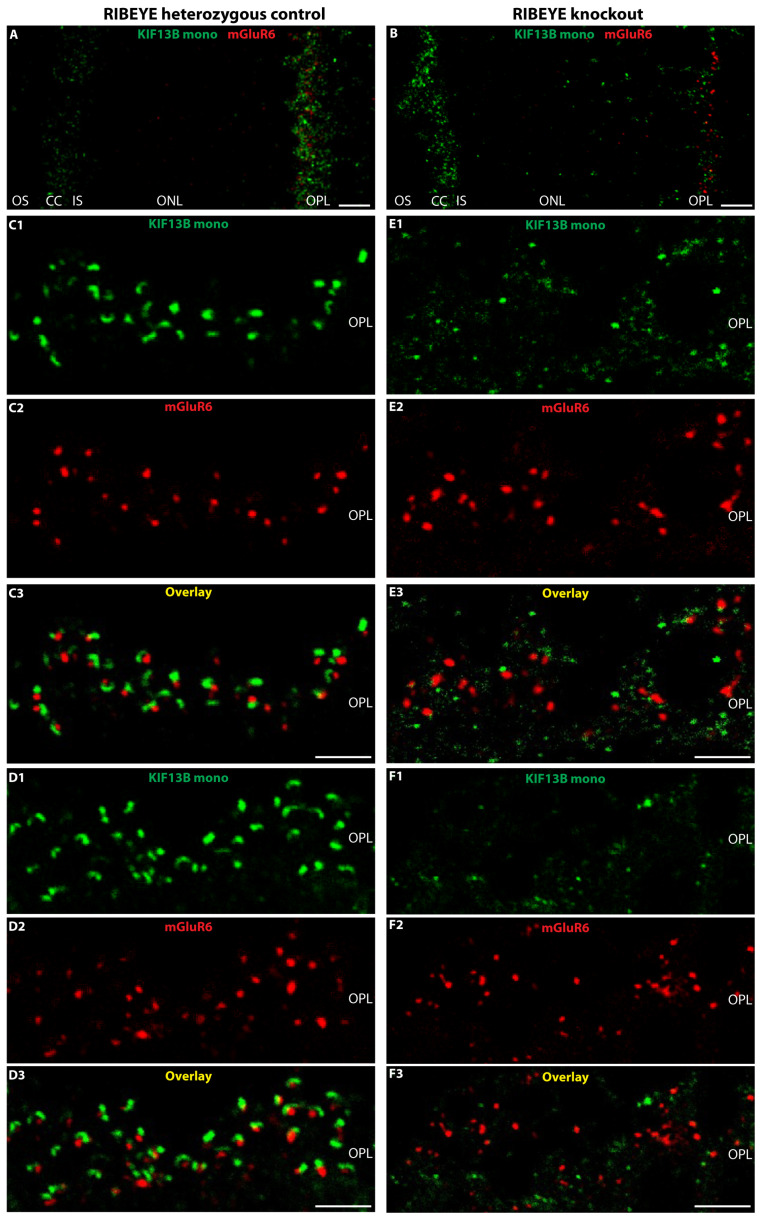
Impact of synaptic ribbon deletion on KIF13B localization in photoreceptor synapses. Cryostat sections of retinas obtained from RIBEYE knockout mice (**B**,**E1**–**E3**,**F1**–**F3**) that completely lack synaptic ribbons [26], and heterozygous control mice (**A**,**C1**–**C3**,**D1**–**D3**) immunolabeled with mouse monoclonal KIF13B (clone 5C10, green channel) and rabbit polyclonal mGluR6 (red channel) to label the dendritic tips of invaginating bipolar cells that are located close to the presynaptic active zone release site. In (**A**,**B**), low magnification micrographs of the indicated immunolabeled sections are presented (as merged images) in which both the connecting cilia (CC) and the photoreceptor synapses in the OPL are visible. In heterozygous control retinas (**A**), the KIF13B immunosignals are stronger in the OPL than at the CC in contrast to the RIBEYE knockout (**B**). Signals from the respective red channels and green channels are merged in (**C3**,**D3**,**E3**,**F3**). Abbreviations: OS, outer segment; CC, connecting cilium; IS, inner segment; OPL, outer plexiform layer; mono, monoclonal. Scale bars: 5 µm.

Since synaptic ribbons are absent in RIBEYE knockout mice [26,28], we used an antibody against mGluR6 (postsynaptic glutamate receptor marker) as a reference protein. mGluR6 is located directly opposite of the presynaptic active zone release sites on the dendritic tips of invaginating rod bipolar cells [63,64,65]. Double immunolabeling analysis with rabbit antibodies against the postsynaptic marker mGluR6 and with mouse monoclonal KIF13B antibody clone 5C10 demonstrated that the synaptic enrichment of KIF13B at the active zone was disturbed by the absence of synaptic ribbon in RIBEYE knockout mice in comparison to the RIBEYE heterozygous mice (Figure 8). In the ribbon-deficient RIBEYE knockout mice, KIF13B was less enriched at the active zone.

RIBEYE knockout mice lack synaptic ribbons in outer plexiform layer (OPL), as well as in the entire retina [26], and an antibody against postsynaptic glutamate receptor mGluR6 was, therefore, used to mark the OPL. The mGluR6 glutamate receptor is expressed at the dendritic tips of the invaginating bipolar sites close to the presynaptic active zone transmitter release sites [63,64,65]. In photoreceptor synapses of RIBEYE knockout mice, the KIF13B signal at the active zone neurotransmitter release site was less strongly enriched at the presynaptic active zones (Figure 8B) and displayed a more diffuse distribution (Figure 8(E1–E3,F1–F3)) than in the heterozygous control retinas (Figure 8A,(C1–C3,D1–D3)) suggesting that the synaptic ribbon is needed for the focal enrichment of KIF13B at the presynaptic active zone.

## 3. Discussion

The main purpose of the present study was to further investigate and to further complete the molecular anatomy of the photoreceptor synaptic ribbon complex. There is strong evidence that RIBEYE is the major protein component of synaptic ribbons [23,24,25,28,29,30,66]. But it is still incompletely understood whether other proteins also contribute to the structure and function of photoreceptor synaptic ribbons.

In the past years, a growing family of ciliary proteins was found to be associated with the photoreceptor synaptic ribbon complex. These proteins were initially discovered as components of microtubule-based photoreceptor primary cilium but were also found at photoreceptor synaptic ribbons. These include the kinesin-2 family protein KIF3A [37,67], the PIP_2_-binding Tubby-like protein1 [68,69,70,71,72], the UNC119 protein [39,73,74] and further ciliary proteins which are involved in the transport of myristoylated proteins [39], as well as the ciliary transition zone protein NPHP4 [36]. Also, proteins of the Bardet–Biedl Syndrome protein complex (BBSome, [75]) of primary cilia were recently reported to be important for photoreceptor synapses and synaptic ribbons [76]. Both photoreceptor primary cilia and photoreceptor synaptic ribbons are compartments with a high activity of vesicle exocytosis [3,9]. Interestingly, vesicle exocytosis at the photoreceptor primary cilium and the photoreceptor ribbon synapse both depend upon the t-SNARE protein syntaxin-3 (instead of syntaxin-1) [77,78,79,80,81,82]. Similarly, photoreceptor-specific knockout of syntaxin-3 not only affects the outer/inner segments but also the synaptic ribbons concomitant with a strong functional impact on phototransduction and synaptic transmission [83].

These previous findings encouraged us to analyze whether the kinesin 3 motor protein KIF13B is also present at the photoreceptor synaptic ribbon complex. KIF13B interacts with NPHP4 in primary cilia of cultured cells [40] and NPHP4 is a component of photoreceptor synaptic ribbons [36]. To address the localization of KIF13B in retinal photoreceptors, we employed two different KIF13B antibodies and light- and electron microscopical immunolabeling techniques. We found a strong enrichment of KIF13B at photoreceptor synaptic ribbons. The enrichment of KIF13B at photoreceptor synaptic ribbons was consistently shown by high-resolution confocal microscopy, super resolution-structured illumination microscopy (SR-SIM) and also at the ultrastructural level by post-embedding immunogold electron microscopy. Furthermore, we demonstrated that the presence of the synaptic ribbon is required for the focal enrichment of KIF13B at the active zone neurotransmitter release sites of photoreceptor synapses. In the absence of synaptic ribbons, as it is the case in the RIBEYE knockout [26], KIF13B was less strongly enriched at the photoreceptor synapse active zone in comparison to the control mice. In the RIBEYE knockout, KIF13B seemed to be more abundant at the photoreceptor connecting cilium than at the synapse. The presynaptic active zone of photoreceptor synapses was indirectly visualized in these experiments by immunolocalization of the postsynaptic mGluR6 metabotropic glutamate receptor. The mGluR6 glutamate receptor is located close to the presynaptic release sites of photoreceptor synapses [63,64,65].

What could be the physiological significance of a growing common set of proteins shared by the photoreceptor primary cilium and the photoreceptor synaptic ribbon? It appears reasonable to assume that identical proteins will perform similar functions at these different subcellular sites. The photoreceptor primary cilium mediates regulated bidirectional transport of proteins from the inner segment into the outer segment and into the opposite direction. The proteins, which are transported into and out of the photoreceptor primary cilium, are important for phototransduction and are involved in light- and dark adaptation [84,85,86]. The synaptic ribbon in the presynaptic photoreceptor terminal promotes continuous synaptic exocytosis by delivering ribbon-associated synaptic vesicles to the active zone release sites [3,19,20,21,22].

Of note, primary cilia enable bidirectional transport, i.e., transport into and out of the primary cilium by making use of the polarity of microtubules. Anterograde transport along the primary cilium is mediated by the kinesin-family of anterograde motor proteins and retrograde transport by the dynein protein-family of retrograde motor proteins [87,88,89,90,91,92,93,94,95,96]. Recently, KIF13B itself was shown to move bidirectionally along the primary cilium [53]. The presence of KIF13B at photoreceptor synaptic ribbons could also possibly suggest such a bidirectional transport system at the synaptic ribbon. So far, only unidirectional vesicle transport has been observed along the synaptic ribbon with synaptic vesicles moving from the cytosolic end of the synaptic ribbon towards the membrane anchored end of the synaptic ribbon that is placed next to the active zone transmitter release site [19,20,21,22,97]. Only indirect evidence is available that could also possibly suggest transport into the opposite (retrograde) direction, i.e., from the membrane-anchored end along the ribbon towards its cytosolic end. This indirect evidence includes the finding that the deletion of the retrograde microtubule motor protein dynein heavy chain has a strong impact on photoreceptor synaptic ribbons [98,99], raising the possibility that retrograde dynein-mediated vesicle trafficking might occur at the synaptic ribbon complex. KIF13B has also been observed to be involved in endocytic events, e.g., endocytosis of LRP1 at caveolin-rich membranes [100,101]. Furthermore, the phosphoinositide-binding protein Tulp1 [102,103] was found in a complex with the photoreceptor synaptic ribbon protein RIBEYE [70] and Tulp1 interacts with dynamin-1, a protein which is crucial for many forms of endocytosis [104]. These findings could indicate retrograde, endocytic vesicle trafficking at the synaptic ribbon complex. But whether these endocytic vesicle trafficking events directly occur on the synaptic ribbon is not clear. Direct evidence for a bidirectional transport along the synaptic ribbon and direct visualization of retrograde transport along the synaptic ribbon is missing according to our knowledge.

In general, the precise role of ATP-consuming microtubule motors at the synaptic ribbon, such as KIF3A [37,67] and KIF13B (this study), for the photoreceptor ribbon synapse is not yet clear. Previous electrophysiological experiments in which intracellular ATP was replaced by non-hydrolyzable ATP analogs (ATP-γS) showed that at a functional level, replenishment and refilling of fusion-competent vesicles was blocked, although previously docked synaptic vesicles could still fuse [105]. At the same time, no morphological alterations of ribbon-associated vesicle pools were observed in the presence of ATP-γS [105]. These findings could indicate that vesicle transport along the synaptic ribbon towards the active zone is blocked in the presence of non-hydrolyzable ATP analogs in a rigor-like manner, thus, preventing the movement of synaptic vesicles along the synaptic ribbon to replenish the active zone with new vesicles after plasma membrane-docked, exocytosis-competent synaptic vesicles have fused with the presynaptic plasma membrane. But other explanations for this phenomenon have also been proposed [105] and theoretical models have been calculated that propose a passive, non-motor-dependent movement of synaptic vesicles along the synaptic ribbon [106].

Knockout mice of the microtubule-dependent kinesin-2 motor KIF3A did not show an obvious morphological phenotype in the photoreceptor presynaptic terminal [107]. Synaptic ribbons were present in the photoreceptor presynaptic terminals of KIF3A knockout mice and were found to be associated with synaptic vesicles with no obvious differences between KIF3A knockout mice and control mice.

Kinesin-3 motor proteins in neurons bind vesicular cargo (and active zone components, e.g., presynaptic neurexins, UNC-10, SYD-2), which is subsequently trafficked into the presynaptic terminal [48,49,50,51,108,109,110,111,112,113,114,115,116,117].

The functional role of KIF13B, a motor protein of the kinesin-3 family [48], has not yet been investigated at the photoreceptor synapse. KIF13B binds to PSD95 [41,118], which is located pre-synaptically in photoreceptor synapses where it scaffolds the presynaptic plasma membrane [119]. Furthermore, KIF13B contains a specialized RPGRIP1N-C2-domain that is important for interaction with NPHP4 [40]. As mentioned, NPHP4 is localized to the photoreceptor synaptic ribbon complex [36]. Early electron microscopic data showed a link between the presynaptic microtubule system and the synaptic ribbon [54]. Furthermore, the plus end of photoreceptor microtubules is located within the presynaptic terminal in which microtubules establish direct contact with the synaptic ribbon [37,54,55,67]. Based on these data, a plus-end-directed anterograde microtubule motor, such as KIF13B, could be involved in the transport of microtubule-bound synaptic vesicles towards the synaptic ribbon. In neuromast hair cells, the microtubule motor KIF1A was found to be important to enrich synaptic vesicles at the synaptic ribbon [120]. Interestingly, KIF13B contains a cytoskeleton-associated protein glycin-rich (CAP-Gly) domain at its Carboxy-terminus [41], which is also present in other microtubule plus end-tracking proteins [121,122,123]. CAP-Gly-domains preferentially bind to tyrosinated tubulins, which are particularly enriched at the +TIPs (plus ends) of microtubules [121,124]. Recently, it was directly shown that KIF13B preferentially binds to tyrosinated microtubules [125]. Thus, KIF13B can be expected to also bind to the +TIPs of microtubules in photoreceptor presynaptic terminals. In support of this notion, the +TIPs of microtubules in neuromast hair cells also point to the basal portion of the cells towards the synaptic ribbons [120].

Interestingly, the ciliary protein NPHP3, which is also localized to photoreceptor synaptic ribbons [39], contains a tubulin-tyrosine ligase (TTL) domain [126]. It has not yet been investigated whether this TTL-domain of NPHP3 is enzymatically active and whether it can tyrosinate α-tubulin in-situ. Clearly, the synaptic ribbon is important for the strong enrichment of KIF13B at the synapse because ribbon-deficient RIBEYE knockout mice did not show a strong enrichment of KIF13B at the active zone. Similar to KIF13B, NPHP3 is also less enriched at the photoreceptor synapse of RIBEYE knockout mice in comparison to control mice [39]. NPHP4 might be involved in the recruitment of KIF13B to the synaptic ribbon because NPHP4 is localized to photoreceptor synaptic ribbons [36] and also binds KIF13B [40]. Further mechanisms could apply. The precise molecular mechanisms of the synaptic enrichment of KIF13B at the synaptic ribbon and its function in the presynaptic terminal remain to be elucidated by future investigations.

## 4. Materials and Methods

### 4.1. Materials

#### 4.1.1. Animals

All experimental procedures that involved mice care or organ dissection were reviewed and approved by the animal welfare and ethics committee of the Saarland University and the local authorities (Landesamt für Verbraucherschutz; GB 3; 66115 Saarbrücken, Germany; GB 3-2.4.1.1-K110/180-07.) Mouse retinas were dissected from C57BL/6J mice within 5 min post-mortem after deep anaesthesia with isoflurane and cervical dislocation, as described [23,39,61]. RIBEYE knockout mice were previously generated and characterized [26]. Mouse genotyping was performed as previously described [26]. Bovine eyes were from a local slaughterhouse and dissected as previously described [23,39]. Eyes from both sexes were used.

#### 4.1.2. Antibodies

##### Primary Antibodies

- anti-KIF13B rabbit polyclonal, antigen affinity-purified (commercial HPA025023, Sigma, Taufkirchen, Germany). The antigen affinity-purified antibody was raised against the peptide LTGKGKLSRRSISSPNVNRLSGSRQDLIPSYSLGSNKGRWESQQDVSQTTVSRGIAPAPA LSVSPQNNHSPDPGLSNLAASYLNP deduced from human KIF13B (amino acid (aa)1369-aa1453; Q9NQT8-1; NM_015254.4, encoding a 1826 aa long protein with a predicted mass of ≈203 kDa) [127]. This peptide stretch is highly conserved in mouse KIF13B (86% amino acid identity in comparison to the human aa sequence) and bovine KIF13B (88% amino acid identities in comparison to the human sequence). This polyclonal KIF13B antibody was used for immunofluorescence (IF) microscopy in a 1:500 dilution, for Western blot (WB) analyses in a 1:1000 dilution and for multi-peptide array WB (“Pepspots“) in a 1:100,000 dilution.
- anti-KIF13B mouse monoclonal: (clone 5C10; IgG2b subtype) was raised against the GST tagged expressed protein of respective KIF13B encoding the sequence stretch KGKLSRRSISSPNVNRLSGSRQDLIPSYSLGSNKGRWESQQDVSQTTVSRGIAPAPALSV SPQNNHSPDPGLSNLAASYLN of mouse KIF13B (aa1371-aa1452 of NM_001081177.3 encoding a 1843 aa long protein) cloned in pGEX-6P-1. The peptide sequence is highly conserved (78% amino acid identities in bovine KIF13B, DAA27057.1; 85% amino acid identities in human KIF13B, EAW63491.1). Cloning of the GST-tagged KIF13B-encoding construct, fusion protein expression and purification, mouse immunization, generation of hybridoma cells, ELISA screening and antibody sub-typing was performed by Absea, Beijing, China. This monoclonal KIF13B antibody was used for IF and EM in a 1:100 dilution, for WB in a 1:1000 dilution and for multi-peptide array WB (“Pepspots”) in a 1:50,000 dilution.
- Additional primary antibodies used in the present study are described in Table 1.

##### Secondary Antibodies

The secondary antibodies used in the present study are given in Table 2.

#### 4.1.3. Plasmids

##### Eukaryotic KIF13B Expression Plasmid

A full-length eukaryotic KIF13B expression clone encoding full-length mouse KIF13B (NM_001081177, 1843 amino acids) was obtained from Genscript Biotech (OMu22296D; Rijswijk, The Netherlands). The full-length mouse KIF13B pcDNA3.1 construct was generated from synthetic DNA with CloneEZ cloning strategy. The sequence of the expression plasmid was verified by DNA sequencing. This full-length KIF13B plasmid was used for the heterologous expression of KIF13B in transfected COS7 cells. Empty pcDNA3.1 plasmid (plasmid without insert) was used for control/mock transfections.

### 4.2. Methods

#### 4.2.1. Preparation of Cryostat Section and Immunolabeling of Cryostat Sections

Cryostat sections of mouse retinas were prepared, as described previously [39,62,132]. In brief, enucleated mouse-eyeball were flash frozen in liquid-nitrogen-cooled isopentane and embedded in Neg-50 frozen section medium (Richard-Allan Scientific, San Diego, CA, USA, 4688521, via Thermo Fisher Scientific, Dreieich, Germany). Bovine retinas were prepared for cryosectioning, as previously described [23,39,62,132]. The bovine eyes were opened along the equator to remove the lens and vitreous body. The retina was peeled off gently by cutting off from the optic nerve. The isolated retina was flash-frozen in liquid nitrogen-cooled isopentane and embedded in Neg-50 frozen section medium. Cryosections (10 µm in thickness) were prepared with a CM950 cryostat (Reichert-Jung/Leica, Wetzlar, Germany). For immunolabeling, cryosections were freshly made from flash-frozen retinas and cryosections were stored at −20 °C until use. Prior to immunolabeling, cryosections were first heat-fixed for 10 min at 60 °C by placing them on a heating pad. After cooling down to room temperature (RT), the immunolabeling of the sections was performed as previously described [23,39,62,132]. Sections were immunolabeled with the primary antibody dilutions described in the material section at 4 °C (ON). After several washes with PBS, sections were incubated with secondary antibodies (1h at RT). Unbound secondary antibodies were removed by rinsing sections with PBS. Finally, the immunolabeled sections were embedded in antifade N-Propyl-gallate (NPG) medium, as previously described [23]. Negative control experiments were performed by omitting the primary antibody. All other steps of the immunolabeling remained unchanged in the negative controls and were performed in parallel with the KIF13B immunolabeling experiments.

#### 4.2.2. Confocal- and Super-Resolution Structured-Illumination Microscopy (SR-SIM)

Immunolabeled cryostat sections were analyzed on a Nikon A1R confocal microscope using 60×/1.40 NA oil objective using the NIS Elements software (NIS Elements AR 3.2, 64bit; Düsseldorf, Germany), as previously described [39,61,70,130,131,132,133,134,135,136,137]. An Elyra PS1 setup (Zeiss, Jena, Germany) equipped with ZEN software (black version) was used to perform Super-Resolution Structured-Illumination-Microscopy (SR-SIM) [39,61,69,133] using a 63× Plan Apo oil objective (N.A. 1.4). Immunolabeling data were confirmed on independent samples from at least three independent animals. Images from RIBEYE knockout and control animals were obtained in a blinded manner with the experimenter not knowing whether the imaging sample was obtained from a knockout mouse or a control mouse.

#### 4.2.3. Antibody Pre-Absorption Control Experiments

- KIF13B (Sigma, Taufkirchen, Germany)

Polyclonal affinity purified KIF13B antibody was diluted 1:1000 in 0.5% BSA in PBS. For blocking, to one half of the antibody dilution, 52 µg of recombinant KIF13B fused with 6-HIS tag expressed fusion protein (APREST76443, Sigma) was added; and 10 µg of p53 protein fused to 6-HIS tag expressed protein (kind gift from Prof. G. Thiel; Department of Biochemistry; Medical School Homburg) as control to the remaining half of the antibody dilution. The fusion protein-antibody mixtures were incubated on a turning wheel overnight (4 °C; ON). On the following day, antibody mixture was centrifuged at 13,000 rpm for 5 min. The pre-absorbed KIF13B antibody was applied on cryostat sections of the bovine retina for IF. Double immunolabeling with mouse monoclonal RIBEYE antibody (clone 2D9) was used as a reference for confocal immunofluorescence (IF) microscopy to label the synapse layers of the retina and to check for possible non-specific blocking effects. Confocal images from pre-absorption control and experimental pre-absorption experiments were acquired at identical conditions using the re-use settings software of NIS Elements (NIS Elements AR 3.2, 64bit; Düsseldorf, Germany). Images were acquired in a blinded manner.

- KIF13B (clone 5C10)

Monoclonal KIF13B antibody was diluted 1:100 in 0.5% BSA in PBS and blocked with the 50 µg of KIF13B GST fusion protein and 25 µg of GST fusion protein The peptide-antibody mixtures were incubated on a turning wheel (4 °C; ON). The pre-absorbed KIF13B antibody was applied on mouse retina cryostat sections for IF. The next day, the antibody mixture was centrifuged at 13,000 rpm for 5 min. The pre-absorbed KIF13B antibody was applied on cryostat sections for IF. Double immunolabeling with rabbit polyclonal RIBEYE antibody (U2656) was used as a reference to label the synapse layers and to check for possible non-specific blocking effects.

#### 4.2.4. Expression and Purification of Recombinant Fusion Protein

Expression and purification of recombinant fusion protein was performed as previously described [23,74]. Recombinant fusion proteins were expressed by electroporation in the electro-competent bacteria BL21(DE3). Transformed bacteria were grown in LB/Ampicillin medium containing 0.5% glucose at 37 °C at 220 rpm until an OD 600 of 0.8–0.9 was achieved. Thereafter, bacterial cultures were induced for protein expression with 0.1 mM IPTG (final concentration) for 5 h at RT and heavy shaking. After the end of the IPTG treatment, all subsequent steps were performed at 4 °C. The bacteria were harvested by centrifugation at 3500 rpm for 20 min at 4 °C. The pellets were washed three times with ice cold PBS by resuspension and centrifugation. After centrifugation, pellets were resuspended in 20 mL of PBS to which 500 µL of 10 mg/mL freshly prepared lysozyme had been added. The bacterial suspension was then incubated for 1 h on ice with gentle agitation. Bacteria were disrupted by sonification (Bandelin Sono Plus, 20 s at 50% power; four repeats). Cell debris was removed by repeated centrifugation (13,000 rpm for 2 h, Thermo Fisher Scientific Biofuge Stratos, Dreieich, Germany). The cell lysate was incubated with pre-swollen glutathione-S-Transferase (GST) agarose beads at 4 °C ON. The GST beads adsorbed to fusion protein were washed thrice with ice cold PBS and sedimented at 1500 rpm for 2 min. The fusion protein was eluted from GST beads by incubating in buffer containing 10 mM reduced glutathione, 50 mM Tris-HCl (pH 8.5) at 4 °C (ON). Protein concentration was determined with Bradford assays [138]. The purity of the fusion protein fractions was assessed by SDS-PAGE.

#### 4.2.5. Post-Embedding Immunogold Labeling

Post-embedding immunogold labeling with the indirect immunogold method was performed largely as previously described [23,39,133,135]. Ultrathin sections from mouse retina were incubated with blocking buffer (0.5% bovine serum albumin (BSA) in PBS) for 1 h at RT to block non-specific protein binding sites. Then, sections were incubated with primary antibody KIF13B mouse monoclonal (clone 5C10) (1:100 dilution) in blocking buffer ON at 4 °C. After several washes with PBS, goat anti-mouse secondary antibody conjugated to 5 nm gold particles (1:100 dilution) in blocking buffer was used to detect binding of KIF13B antibody for 1 h at RT. Next, after several washes with the PBS, 2.5% glutaraldehyde in PBS was used to fix the immune complexes (15 min, RT). Then, sections were gently washed with water and contrasted with 2% uranyl acetate in water for 15 min at RT. Grids were washed several times with water before grids were air-dried. As a negative control, primary antibody was omitted from the procedure with all other steps remaining the same. Sample grids were analyzed with a Tecnai Biotwin 12 digital microscopy (FEI/Thermo Fisher Scientific). Images were acquired with a Megaview III digital camera (Olympus, Hamburg, Germany), controlled by the iTEM software (Olympus, Hamburg, Germany; version 5.0). As a negative control experiment, the primary antibody was omitted with the remaining procedure being the same and were performed in parallel with the experimental KIF13B immunolabeling experiments. Immunolabeling data were confirmed on independent samples from three independent animals.

#### 4.2.6. Multi-Peptide Arrays (“Pepspots”)

For antibody epitope mapping, peptides for aa 1369-aa1453 of human KIF13B and mouse KIF13B (aa 1368-aa 1453) were synthesized on a hardened cellulose membrane with a ResPepSL-Synthesizer (Intavis Bioanalytical Instruments, Cologne, Germany), as previously described [39,139]. The synthesized peptide was 20 aa long in each row with 10 aa overlaps. The membrane was activated with methanol for 1 min and washed twice with water. Then, the membrane was equilibrated with binding buffer (50 mM Tris-HCl, pH 7.5, 150 mM NaCl, 0.1% Triton X-100) for 2 h at RT. Membrane was then blocked with blocking buffer (1 µM BSA in 50 mM Tris-HCl, pH 7.5, 150 mM NaCl) for 1 h at RT. Primary antibody KIF13B (rabbit polyclonal) was incubated at a dilution of 1:100,000 and KIF13B (mouse monoclonal clone 5C10) at a dilution of 1:50,000 (ON at 4 °C). On the next day, the membrane was washed with binding buffer (three times, 5 min each, RT). Then, the secondary antibody (goat anti-rabbit/or goat anti-mouse antibody dilution conjugated to peroxidase, respectively, 1:10,000 dilution) was added to the cellulose membrane and the membrane was incubated in the secondary antibody dilution for 1 h under gentle agitation. The membrane was washed several times with binding buffer to remove unbound antibody. The ECL signals were documented with a Chemidoc™ XRS Gel Doc (Bio-Rad, Feldkirchen, Germany) apparatus. The location of the individual peptide spots in the multi-peptide arrays was visualized by UV illumination.

#### 4.2.7. Heterologous Protein Expression in COS7 Cells

COS7 cells were cultured in DMEM supplemented with 10% fetal calf serum (FCS). KIF13B eukaryotic expression plasmid was transfected transiently in COS cells using PerFectin transfection reagent (Genlantis/AMSBIO, Frankfurt am Main, Germany) for 48 h (37 °C, 5% CO_2_). Transfection was performed according to the manufacturer’s instructions. The cell lysates were prepared using lysis buffer (100 mM Tris-HCl, pH 8.0, 150 mM NaCl, 1 mM EDTA and 1% Triton X-100). The cell lysate was centrifuged at 13,000 rpm for 10 min. Protein concentration of the supernatant was determined by Bradford assay [138]. A total of 50 µg of total protein was boiled in Laemmli SDS sample buffer, loaded on 7% acrylamide (AA) SDS-PAGE and analyzed with WB analyses, as described below.

#### 4.2.8. SDS-PAGE and Western Blot

SDS-PAGE and Western blotting experiments were performed as previously described [23,39,61,135]. The dissected mouse retinas were incubated with about 70 µL lysis buffer per retina (0.1% Triton X-100 in PBS, pH 7.4) for 30 min on ice and samples were homogenized with brief sonication at 20% power. Each single dissected bovine retina was lysed in a total volume of 1 mL of lysis buffer for 30 min on ice. The retinal lysate was centrifuged for 10 min at 13,000 rpm and SDS Laemmli buffer was added to the supernatant (approx. in a 1:1 volume ratio). Samples were boiled for 5 min and subjected to 7% acrylamide SDS PAGE gel. Roti standard marker was used to determine the running position/band size of respective proteins. Proteins were transferred to nitrocellulose membrane (Protran 0.45 µm) at 50 V, 6 h, 4 °C. The nitrocellulose membrane was incubated with 5% skimmed milk powder in PBS to block non-specific binding sites followed by overnight incubation in primary antibody at 4 °C at the dilutions given in the Materials section. The peroxidase-conjugated secondary antibodies were used for detecting the binding of the respective primary antibodies as indicated in Table 2. The signals were analyzed with ECL using ChemiDoc™ XRS Gel Doc system (Bio-Rad).

For the re-probing of nitrocellulose membranes with additional antibodies, membranes were “stripped” by incubation in stripping buffer (100 mM Tris, pH 7, 0.2% SDS, 80 mM β-Mercaptoethanol; pre-warmed to 60 °C) for 10 min at RT.

## Figures and Tables

**Table 1:** Additional primary antibodies used in the study.

Primary Antibodies	References/Source	Dilution
anti-RIBEYE mouse monoclonal antibody (2D9) against the C-terminus of RIBEYE(B)-domain/CtBP2	[28,61]	1:1000 (IF) 1:1000 (WB) 1:1000 (EM)
anti-actin (clone C4) mouse monoclonal antibody	Millipore, Burlington, MA, USA, MAB1501 [128]	1:1000 (WB)
anti-RIBEYE rabbit polyclonal antibody (U2656) against RIBEYE(B)-domain	[23]	1:1000 (IF)
anti-PSD95 (post-synaptic density protein 95) rabbit polyclonal antibody (L667)	[129]	1:1000 (IF)
anti-mGluR6 rabbit polyclonal antibody (1205)	[26,130]	1:2000 (IF)
anti-Cav1.4 Cterm rabbit polyclonal antibody	[131]	1:1000 (IF)

Abbreviations: IF, immunofluorescence microscopy; EM, immunogold electron microscopy; Pepspot, multi-peptide array; WB, Western blot.

**Table 2:** Secondary antibodies.

Antibody	Source	Dilution
Chicken anti-mouse Alexa488	Invitrogen Molecular Probes, Eugene, OR, USA, A-21200	1:1000 (IF)
Donkey anti-mouse Alexa488	Invitrogen Molecular Probes, A-21202	1:1000 (IF)
Donkey anti-rabbit Alexa568	Invitrogen, Molecular Probes, A-10042	1:1000 (IF)
Chicken anti-rabbit Alexa488	Invitrogen, Molecular Probes, A-21441	1:1000 (IF)
Donkey anti-mouse Alexa568	Invitrogen, Molecular Probes, A-10037	1:1000 (IF)
Goat anti-mouse Alexa647	Invitrogen, Molecular Probes, A-21236	1:1000 (IF)
Donkey anti-mouse Alexa647	Invitrogen, Molecular Probes, A-31571	1:1000 (IF)
Goat anti-rabbit peroxidase-conjugated (POX) IgG	Sigma, A-6154	1:3000 (WB) 1:10,000 (Pepspots)
Goat anti-mouse peroxidase-conjugated (POX) IgG	Sigma, A-3673	1:3000 (WB) 1:10,000 (Pepspots)
Goat anti-mouse secondary antibody conjugated to 5 nm gold particles	Sigma, G7527	1:100 (EM)

Abbreviations: IF, immunofluorescence microscopy; EM, immunogold electron microscopy; Pepspot, multi-peptide array; WB, Western blot.

## Data Availability

Data is contained within the article; further inquiries can be directed to the authors.
